# Immediate versus delayed treatment for recently symptomatic carotid artery stenosis

**DOI:** 10.1590/1516-3180.20161346T2

**Published:** 2016-06-03

**Authors:** Vladimir Vasconcelos, Nicolle Cassola, Edina Mariko Koga da Silva, José Carlos Costa Baptista-Silva

## Abstract

**BACKGROUND:**

The timing of surgery for recently symptomatic carotid artery stenosis remains controversial. Early cerebral revascularization may prevent a disabling or fatal ischemic recurrence, but it may also increase the risk of hemorrhagic transformation, or of dislodging a thrombus. This review examined the randomized controlled evidence that addressed whether the increased risk of recurrent events outweighed the increased benefit of an earlier intervention.

**OBJECTIVES:**

To assess the risks and benefits of performing very early cerebral revascularization (within two days) compared with delayed treatment (after two days) for people with recently symptomatic carotid artery stenosis.

**METHODS:**

*Search methods:* We searched the Cochrane Stroke Group Trials Register in January 2016, the Cochrane Central Register of Controlled Trials (CENTRAL; The Cochrane Library 2016, issue 1), MEDLINE (1948 to 26 January 2016), EMBASE (1974 to 26 January 2016), LILACS (1982 to 26 January 2016), and trial registers (from inception to 26 January 2016). We also handsearched conference proceedings and journals, and searched reference lists. There were no language restrictions. We contacted colleagues and pharmaceutical companies to identify further studies and unpublished trials

*Selection criteria:* All completed, truly randomized trials (RCT) that compared very early cerebral revascularization (within two days) with delayed treatment (after two days) for people with recently symptomatic carotid artery stenosis.

*Data collection and analysis:* We independently selected trials for inclusion according to the above criteria, assessed risk of bias for each trial, and performed data extraction. We utilized an intention-to-treat analysis strategy.

**MAIN RESULTS:**

We identified one RCT that involved 40 participants, and addressed the timing of surgery for people with recently symptomatic carotid artery stenosis. It compared very early surgery with surgery performed after 14 days of the last symptomatic event. The overall quality of the evidence was very low, due to the small number of participants from only one trial, and missing outcome data. We found no statistically significant difference between the effects of very early or delayed surgery in reducing the combined risk of stroke and death within 30 days of surgery (risk ratio (RR) 3.32; confidence interval (CI) 0.38 to 29.23; very low-quality evidence), or the combined risk of perioperative death and stroke (RR 0.47; CI 0.14 to 1.58; very low-quality evidence). To date, no results are available to confirm the optimal timing for surgery.

**AUTHORS CONCLUSIONS:**

There is currently no high-quality evidence available to support either very early or delayed cerebral revascularization after a recent ischemic stroke. Hence, further randomized trials to identify which patients should undergo very urgent revascularization are needed. Future studies should stratify participants by age group, sex, grade of ischemia, and degree of stenosis. Currently, there is one ongoing RCT that is examining the timing of cerebral revascularization.

The full text of this review (English), the abstract (English and Polish) and the plain language summary (English and Polish) are available from: http://onlinelibrary.wiley.com/wol1/doi/10.1002/14651858.CD011401.pub2/abstract
